# Should we consider assisted suicide a clinically and ethically valuable option for people with mental disorders? Con Speaker

**DOI:** 10.1192/j.eurpsy.2025.41

**Published:** 2025-08-26

**Authors:** J. Samochowiec

**Affiliations:** Deptartment of Psychiatry, Pomeranian Medical University, Szczecin, Poland

## Abstract

**Abstract:**

Several challenges exist rendering people with mental disorders particularly vulnerable to wrongful assisted suicide. Their desire to die may be a symptom of the mental illness rather than an autonomous choice; their decision-making competence may be compromised by the illness and hence more troublesome to determine; the severity of suffering may be more challenging to assess from an external perspective; the very wish to die may be variable over time; and prognostic uncertainty in mental illness may impede determining of whether severe suffering is, in fact, resistant to treatment. An ethically sound argument for excluding people with mental disorders from assisted suicide is their potential inability to make a free, autonomous decision. A person’s request for assisted suicide should be considered in the context of an assessment of their capacity to make a well-informed and deliberated decision. Opponents of legalising euthanasia and assisted suicide agree that the suffering caused by mental illness can be just as severe and agonising as in cases of somatic conditions. However, one cannot with all certainty assume that the illness is incurable and that the suffering cannot be minimised to such an extent that the patient experiences relief. The task of psychiatry is to prevent suicide. What follows is that diseases such as depression should be treated, while the patients should be supported and not facilitated to part with their lives. Considering their potentially illness-affected request for suicide, it seems better to prolong their suffering than let them die. One cannot grant the right to die to all those who have the competence to make decisions. Furthermore, legalising euthanasia and assisted suicide also in the case of patients with mental illnesses risks distorting the doctor-patient relationship. Euthanasia is not a balance between the interest of the state and the benefit of the individual in terms of choosing the time and manner of their death. What happens if the interest of the state is not to protect the lives of citizens, especially the most vulnerable and defenceless, including the mentally ill? Once values are rejected, only practical **‘**benefits’ remain, such as financial savings on psychiatric care and treatment. And this is extremely risky. Drawing a clear line in the light of the above considerations seems to be impossible. In the current situation, where many people are exposed to mental suffering, e.g. in the form of depression, the priorities are suicide prevention policies and funding programmes for psychiatric treatment. The debate will take into account arguments for and against euthanasia in patients with mental disorders.

**Disclosure of Interest:**

None Declared

